# Body Language Analysis in Healthcare: An Overview

**DOI:** 10.3390/healthcare10071251

**Published:** 2022-07-04

**Authors:** Rawad Abdulghafor, Sherzod Turaev, Mohammed A. H. Ali

**Affiliations:** 1 Department of Computer Science, Faculty of Information and Communication Technology, International Islamic University Malaysia, Kuala Lumpur 53100, Malaysia; 2 Department of Computer Science and Software Engineering, College of Information Technology, United Arab Emirates University, Al-Ain, Abu Dhabi P.O. Box 15556, United Arab Emirates; 3 Department of Mechanical Engineering, Faculty of Engineering, University of Malaya, Kuala Lumpur 50603, Malaysia

**Keywords:** body language, pandemic, epidemic, body language analysis, AI

## Abstract

Given the current COVID-19 pandemic, medical research today focuses on epidemic diseases. Innovative technology is incorporated in most medical applications, emphasizing the automatic recognition of physical and emotional states. Most research is concerned with the automatic identification of symptoms displayed by patients through analyzing their body language. The development of technologies for recognizing and interpreting arm and leg gestures, facial features, and body postures is still in its early stage. More extensive research is needed using artificial intelligence (AI) techniques in disease detection. This paper presents a comprehensive survey of the research performed on body language processing. Upon defining and explaining the different types of body language, we justify the use of automatic recognition and its application in healthcare. We briefly describe the automatic recognition framework using AI to recognize various body language elements and discuss automatic gesture recognition approaches that help better identify the external symptoms of epidemic and pandemic diseases. From this study, we found that since there are studies that have proven that the body has a language called body language, it has proven that language can be analyzed and understood by machine learning (ML). Since diseases also show clear and different symptoms in the body, the body language here will be affected and have special features related to a particular disease. From this examination, we discovered that it is possible to specialize the features and language changes of each disease in the body. Hence, ML can understand and detect diseases such as pandemic and epidemic diseases and others.

## 1. Introduction

Body language constitutes one of the languages of communication. The types of languages are classified into verbal and non-verbal languages. Body language includes non-verbal language, where the movements and behaviors of the body are used instead of words to express and convey information. Body language may involve hand movements, facial expressions and hints, eye movements, tone of voice, body movements and positions, gestures, use of space, and the like. This research will focus on interpretations of the human body language, classified under kinesiology.

Body language is entirely different from sign language, a complete language—like verbal language—with its own basic rules and complex grammar systems [1,2]. On the other hand, body language does not contain grammatical rules and is usually a language belonging to or classified according to cultures [3]. Interpretations of body language may differ from country to country and from one culture to another. There exists some controversy over whether body language can be regarded as a universal language for all people. Some researchers have concluded that most communication among individuals involves physical symbols or gestures since the interaction of body language here facilitates speedy information transmission and understanding [4]. According to [5], body language speaks more and better content than verbal language. When, for example, an individual speaks over the phone to someone about an inquiry, the information becomes mysterious due to the physical language’s restrictions. However, an individual sitting directly in front of an audience has fewer restrictions and does not have an audience. The information with body language is more easily transmitted and received, even more so if the speaker is standing, allowing more freedom of movement. Thus, it follows that body language enhances communication. This work attempts to prove that body language enhances workplace positivity.

Several experiments were performed in [6] on facial expressions and body movements affected by human emotions. The study has shown that facial expressions and body movements can accurately determine human emotions. It also proved that combining facial features and activities with body movements is essential for analyzing human expressions. Three different stages of experiments were conducted to determine whether it is necessary to combine the two expressions or not. It was confirmed that it is essential to connect them for identification. Reading someone’s eyes should also not be ignored. It is considered an important factor in expressing and understanding human emotions. We are generally able to know what others want from their eye movements. For that, eye language has many effects. According to [7], the expansion and tightness of the eye size are affected by emotions and allow the observer to convey specific additional information. The human eye blinks, on average, 6 to 10 times per minute. However, when someone is attracted to someone else, the number of blinks is fewer. Study [8] discovered that human feelings could be identified and defined through body position. For example, when a person feels angry, they will push their body forward to express dominance over the other person, and their upper body is tilted and no longer upright. On the other hand, if someone feels intimidated by the opponent, they signal submission by retreating backward or moving their head back. Additionally, a person’s emotional state can be determined from their sitting position. Someone sitting on a chair with half of their upper body and head slightly tilted forward indicates attentiveness and eagerness to follow what is being said. However, sitting with legs and hands crossed suggests that they do not wish to engage and feel uncomfortable with what is being said or the person saying it [5].

Body language analysis is also essential to avoid confusion in a single movement’s meanings and purposes that carry more than one meaning. For example, the expressive movement of a person may be due to a physical handicap or a compulsive movement rather than an intentional one. Furthermore, a particular movement in the body of someone may not mean the same to another. For example, a person may rub their eyes due to itchiness and not fatigue. Foreign cultures also need careful analysis due to their social differences. Although most body movements are universal, there are also movements specific to each culture. This may vary from country to country, region to region, and even social group.

Pandemic and epidemic diseases constitute a global risk factor responsible for the death of millions of people worldwide. The ability to detect and treat casualties is limited, primarily due to the lack of human and technical resources. When patients are not physically accessible, remote diagnosis is required. All pandemic and epidemic diseases are characterized by distinct body movements affecting the face, shoulders, chest, and hands. AI technology has shown positive results in some reading of these gestures. Hence, the idea is to use body language to detect epidemic diseases early and provide treatment. It should be noted that the primary and vital catalyst for the proposal of this study is the COVID-19 disease, which is presently terrorizing the whole world. As researchers in information technology and computer science, we must play our part in rapidly detecting this disease.

This paper aims to study the previous literature and identify body language expressions that indicate disease. Body language is defined as certain expressions, movements, and gestures that point to the physical and emotional state of the bearer. Certain parts of the body can express different characteristics or feelings. Some studies have demonstrated the presence of certain emotional conditions as reflected in particular facial expressions (e.g., joy, sadness, surprise, and anger). Regarding the relationship between diseases and body language, it is known that diseases affect the body parts and qualities and are reflected in the movements and expressions of parts of the body. Different diseases affect different body parts and can be measured, identified, and used for diagnosis.

Hence, this paper is proposed to study some diseases that can be diagnosed by identifying and measuring the external movements of the body. In addition, this paper discusses the findings of previous studies to demonstrate the usefulness and contribution of AI in detecting diseases through body language. One of the biggest obstacles to treating COVID-19 patients effectively is speedy diagnosis. However, the large number of cases exceeds the capacity of most hospitals. Hence, AI offers a solution through ML. ML can detect disease symptoms as manifested in the patient’s body language and can be used to generate correct readings and predictions.

Therefore, the main contribution of this paper is to show the potential use of analyzing body language in health care. The importance of body language analysis in health care and patient body language analysis using AI will be discussed in the following sections. The added tables list previous studies that used ML to identify symptoms through body expressions. The findings demonstrate that a patient’s body language can be analyzed using ML for diagnostic purposes.

## 2. Methodology

**Figure 1:**
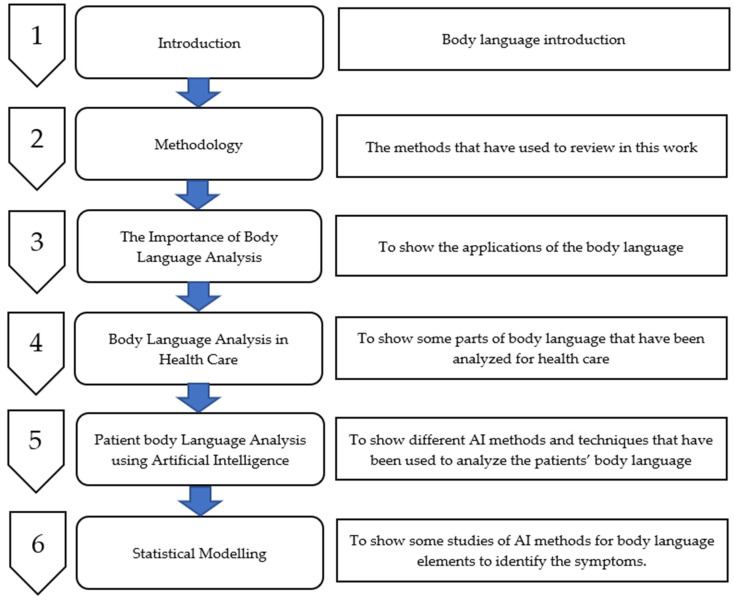
The Review Stages.

The methods used to review in this work are as follows (also see Figure 1): first, the importance of body language analysis is highlighted to prove that the body movements can be read and analyzed to produce outcomes that are useful for many applications; second, body language analysis in health care is presented to show the importance of body language in medical diagnosis in research; third, ML is used successfully to identify characteristic symptoms; fourth, Table 1 show studies that used ML as a diagnostic tool and include the used algorithms. Each topic was discussed separately, as detailed in the following sections.

## 3. The Importance of Body Language Analysis

AI is one of the most significant technological developments, increasing in popularity and being used in all application areas. One of the most important of these applications is the use of AI in healthcare. Health is the most important human factor for life on this planet. Recently, the use and applications of AI in healthcare have played a significant role in helping doctors discover diseases and improve human health. The use of AI in health depends on the appearance of some symptoms on parts of the body. These symptoms affect and are reflected in the movements and expressions of the body, which are manifested as body language. From this point, these features of body language can be used to classify disease symptoms by detecting them in ML. In this section, we want to explain the importance of using body language by artificial intelligence. There are features that appear in body language that AI can analyze to solve many problems in many applications. For example, facial expressions can be analyzed to know human feelings and benefit from them in psychotherapy or examine subjects’ emotions in the study. Another example is analyzing the movements of the hand, shoulder, or leg, and using them to discover valuable features in medicine, security, etc. From this point, we want to show that body language has many benefits and applications, so this is important. Therefore, we want to suggest that body language can also be used to detect infectious diseases such as COVID-19 using ML.

Now, it is feasible to employ this technology in healthcare systems. Pandemic and epidemic diseases are considered an intractable matter that inferiorly affects human health, regarded as peoples’ most valuable asset. Additionally, the biggest worry is that new pandemics or epidemics will suddenly appear and become deadly, such as COVID-19, which has claimed nearly a million lives so far. This stimulates us to develop AI technologies to help detect the disease’s external symptoms by analyzing the patients’ body language. This work deals with general studies that prove the importance of body language processing in various fields.

Every computer user interacts with the device via mouse and keyboard. Currently, researchers are developing a computer system for interaction and response through body language such as hand gestures and movement. In [8], a comprehensive survey was completed evaluating the published literature recommending the visual interpretation of hand gestures when interacting with computing devices and introducing more advanced methods to analyze body language rather than mouse and keyboard movements. The study of [9] considered the problem of robot accuracy recognition. It proposed a fusion system to identify the fall movement types and abnormal directions with an accuracy rate of 99.37%. A facial coding system was developed in [10] to measure and analyze facial muscle movements and identify facial expressions. A database was created with a series of 1100 images. The system analyzed and classified facial creases and wrinkles to match their movements. The results showed that the performance improved, reaching 92%. Combining facial features and movements with body movements is essential for analyzing individual expressions. Three different experiments were conducted to determine whether facial expressions and body language should be combined and concluded in the affirmative. Another study [11] focused on deep learning techniques to identify emotions revealed in facial expressions. This research used pure convolutional neural network techniques to prove that deep learning using these neural networks successfully recognizes emotions by developing cognition, significantly improving the usability. A new model was invented in [12] that detected body gestures and movements with a pair of digital video images, which supplied a set of vector monitors with three dimensions.

The first study showed the relationship between the contraction of the internal muscles of the face and the facial movements as established by Hjortsjo 1970 [13] to develop a coding system by identifying the minor units of facial muscle movements and then drawing coordinates that defined the facial expressions. The recognition of people’s emotions has merited much attention. However, the issue of detecting facial emotions and expressions of speech, especially among researchers, is still problematic. The work presented in [14] offered a comprehensive survey to facilitate further research in this field. It focused on identifying gender-specific characteristics, setting an automatic framework to determine the physical manifestation of emotions, and identifying constant and dynamic body shape comments. It also examined recent studies on learning and emotion by identifying gestures through photos or video. Several methods combined speech, body, and facial gestures were also discussed to identify optimized emotions. The study concluded that the knowledge of a person’s feelings through overtones was still incomplete.

## 4. Body Language Analysis in Healthcare

A coding system was created to classify the facial expressions by analyzing more than 1100 pictures at work [10]. Three ways to classify facial expressions were compared: a method for analyzing image components in the gray field, measuring wrinkles, and a template for creating facial movements. The accuracy of performance of the coding system for the three roads was 89%, 57%, and 85%, respectively, while when assembling the methods, the performance accuracy reached 92%. Online learning is challenged by knowing students’ participation in learning processes. In work [15], an algorithm is introduced to learn about student interactions and see their problems. In this algorithm, two methods were used to collect evidence of student participation: the first method involved collecting facial expressions using a camera, and the second involved collecting hand movement data using mouse movements. The data were trained by building two groups; one group collected facial data with mouse data, and the second was without the mouse. It was discovered that the first group’s performance was better than the second group’s by 94.60% compared to 91.51%. Work [14] commented on recognizing facial and speech gestures that may provide a comprehensive survey of body language. It provided a framework for the automatic identification of dynamic and fixed emotional body gestures that combined facial and speech gestures to improve recognition of a person’s emotions. Paper [16] defines facial expressions by matching them with body positions. The work demonstrated that the effects and expressions are more evident when the major irritations on the face are similar to those highlighted in the body. However, the model produces different results according to the dependence on the properties, whether physical, dimensional, or latent. Another significant finding in the study is that expressions of fear bloom better when paired with facial expressions than when performing tasks.

In [17], the authors stated that the medical advisor must exhibit exciting communication qualities that make the patient feel comfortable making a correct decision. They advised doctors to know how to use facial expressions, eyes, hand gestures, and other body expressions. It was mentioned that a smile is the most robust expression that a doctor can use to communicate with their patients, as the doctor’s smile makes the patient feel comfortable. The patient’s sense of comfort makes them appear confident, and they answer the doctor’s questions with clear responses, credibility, and confidence. In addition, communicating with the eyes is very important to help the patient, as the lack of this from the doctor may suggest that the doctor does not care about them. The research in [18] concludes that the doctor’s appropriate nonverbal communication positively impacts the patient. Objective evidence has shown that the patient improves and recovers better and faster when the doctor uses a smile and direct eye communication with the patient compared to those who do not use a smile and direct eye with the patient. It was also concluded that patients who receive more attention, feeling, sensation, and participation by the doctor respond better to treatment, as the tone of voice, movement of the face and body, and eye gaze affect the patient. Clint [19] reported his first day on the job in the intensive care unit. He felt fear and anxiety on that day as the unit was comprehensive and informative. Clint was asking himself, “is it worth working in that unit?” He had a patient with her sister next to her. The patient glimpsed Clint’s nervousness and anxiety but did not dare ask him, so she whispered that the nurse was nervous to her sister. Then, her sister asked Clint, “you are worried and anxious today; why?” What is there to be so nervous about? Clint thought to hide his nervousness and anxiety and restore confidence; he smiled and replied, “I am not nervous.” However, sometimes, we have to ask our patients ridiculous questions that make us tense. Here, Clint states that he noticed from the patient’s looks that he could not persuade her to hide his stress. Clint made it clear that patients are affected by their body language and facial expressions. They can know their cases through their body language. From here, Clint realized that he was wrong. As anxiety and stress began on his patient, his condition may increase for that reason.

In one of Henry’s articles [20], he wrote that treating a patient with behaviors and body language has a more significant impact than using drugs. The work [21] concluded that non-verbal language between a doctor and their patient plays a vital role in treating the patient. The doctor can use non-verbal signals sent from the patient to collect information about the condition of the disease to help them decide on diagnosis and treatment. The research summarized that the non-verbal technique used by the doctor toward the patient affects them in obtaining information and helping them recover from the disease. For example, eye gaze, closeness to the patient, and facial and hand gestures to appear relaxed. The research suggests that there is a positive effect on the use of non-verbal cues on the patient. It is recommended that doctors be trained in incorporating non-verbal cues as a significant way of dealing with patients to speed up their treatment.

## 5. Patient’s Body Language Analysis Using AI

Different AI methods and techniques have been used to analyze patients’ body language. We briefly discuss some studies conducted so far in this area. More specifically, focusing on facial recognition, a pimple system was introduced in [22] to analyze facial muscles and thus identify different emotions. The proposed system automatically tracks faces using video and extracts geometric shapes for facial features. The study was conducted on eight patients with schizophrenia, and the study collected dynamical information on facial muscle movements. This study showed the possibility of identifying engineering measurements for individual faces and determining their exact differences for recognition purposes. Three methods were used in [23] to measure facial expressions to define emotions and identify persons with mental illness. The study’s proposed facial action coding system enabled the interpretation of emotional facial expressions and thus contributed to the knowledge of therapeutic intervention for patients with mental illnesses.

Many people suffer from an imbalance in the nervous system, which leads to paralysis of the patient’s movement and falls without prior warning. The study [24] was targeted to improve early warning signs detection and identification rate using a platform (R). Wireless sensor devices were placed on the chest and waist. The collected data were converted to an algorithm for analysis that extracted them and activated if there was a risk. The results showed that the patient at risk engaged in specific typical movements, which indicated an imminent fall. The authors further suggested applying this algorithm to patients with seizures to warn of an imminent attack and alert the emergency services.

In research [25], a computational framework was designed to monitor the movements of older adults to signal organ failures and other sudden drops in vital body functions. The system monitored the patient’s activity and determined its level using sensors placed on different body parts. The experiments show that this system identifies the correct locations in real-time with an accuracy of 95.8%. Another approach based on data analysis was presented in [26] for an intelligent home using sensors to monitor its residents’ movements and behaviors. This system helps detect behaviors and forecast diseases or injuries that residents may experience, especially older people. This study is helpful for doctors in providing remote care and monitoring their patients’ progress. The target object capture setup model proposed in [27] is based on the candidate region–suggestion network to detect the position grab of the manipulator combined with information for color and deep image capture using deep learning. It achieved a 94.3% crawl detection success rate on multiple target detection datasets through merging information for a color image. A paper [28] under review deals with the elderly and their struggle to continue living independently without relying on the support of others—the research project aimed to compare automated learning algorithms used to monitor their body functions and movements. Among the eight higher education algorithms studied, the support conveyor algorithm achieved the highest accuracy rate of 95%, using reference traits. Some jobs require prolonged sitting, resulting in long-term spinal injury and nervous disease. Some surveys helped design sitting position monitoring systems (SPMS) to assess the position of the seated person using sensors attached to the chair. The drawback of the proposed method was that it required too many sensors. This problem was resolved by [29], who designed an SPMS system that only needed four such sensors. This improved system defined six different sitting positions through several machine-learning algorithms applied to average body weight measurements. The positions were then analyzed and classified into any approach that would produce the highest level of accuracy, reaching from 97.20% to 97.94%. In most hospitals, medical doctors face anxiety about treating patients with mental illness regarding potential bodily harm, staff risks, and hospital tool damage. The study [30] devised a method to analyze the patient’s movements and identify the risk of harmful behavior by extracting visual data monitoring the patient’s movements from cameras installed in their rooms. The proposed method traced the movement points, accumulated them, and extracted their properties. The characteristics of the movement points were analyzed according to spacing, position, and speed. The study concluded that the proposed method could be used to explore features and characteristics for other purposes, such as analyzing the quality of the disease and determining its level of progression. In the study [31], wireless intelligent sensor applications and devices were designed to care for patient health, provide better patient monitoring, and facilitate disease diagnosis. Wireless sensors were installed on the body to periodically monitor the patient’s health, update the information, and send it to the service center. The researchers investigated the multi-level decision system (MDS) to monitor patient behaviors and match them with the stored historical data. This information allowed the decision makers in the medical centers to give treatment recommendations. The proposed system could also record new cases, store new disease data, and reduce the doctors’ effort and time spent examining the patients. The results proved accurate and reliable (MDS) in predicting and monitoring patients.

The study of [32] proposed the Short Time Fourier Transform application to monitor the patient’s movements and voice through sensors and microphones. The system transmitted sound and accelerometer data, analyzed the data to identify the patient’s conditions, and achieved high accuracy. Three experiments were conducted in reference [33], which involve the recognition of full-body expressions. The first experiment was about matching body expressions to incorporate all emotions, where fear was the most difficult emotion to express. At the same time, the second experiment focused on facial expressions strongly influenced by physical expression and, as a result, was ambiguous. In the last experiment, attention was given to expressions of the tone of a voice to identify emotional feelings related to the body. Finally, it was concluded that it was essential to pool the results of the three experiments to reveal true body expression.

A valuable study was conducted at the MIT Institute [34] to develop a system that detects pain in patients by analyzing data on brain activities using a wearable device to scan brain nerves. This was shown to help diagnose and treat patients with loss of consciousness and sense of touch. In this research, researchers use several fNIRS sensors specifically on the patient’s front to measure the activity of the frontal lobe, where the researchers developed ML models to determine the levels of oxygenated hemoglobin related to pain. The results showed that pain was detected with an accuracy of 87%.

The study [35] considered the heartbeat as a type of body language. Checking a patient’s heartbeat constitutes a crucial medical examination tool. The researcher suggested a one-dimensional (1D) convolutional neural network model CNN, which classified the vibrational signals of the regular and irregular heartbeats through an electrocardiogram. The model used the de-noising auto-encoder (DAE) algorithm, and the results showed that the proposed model classified the sound signals of the heart with an accuracy of up to 99%.

## 6. Discussion

We can conclude from this study that reading and understanding body language through AI will help automatically detect epidemic diseases. Counting epidemic patients is a significant obstacle to detecting every infected person. The most prominent example that is evident now is COVID-19 sufferers. All the developed, middle, and developing countries of the world have faced a significant problem examining the disease due to many infected people and the rapid spread. Thus, infections increased significantly, making it difficult to catch up to detect. We suggest conducting a study to determine the movements and gestures of the body with epidemic diseases, such as those with COVID-19. Indeed, the epidemic disease will have unique and distinct movements in some body parts. The thermal camera to detect high body temperature certainly plays a significant role in indicating a patient with a disease. Still, it is difficult to determine what kind of disease is affected, and secondly, there may be a patient with epidemic disease, but their temperature may not have significantly increased. Thirdly, it may be revealed that the high temperature of an epidemic may be delayed, and the patient is in a critical stage of treatment. We focus in this study on the interest in studying the body language of some epidemics, especially COVID-19, which changed our lives for the worse. We have learned a harsh lesson from this deadly enemy: not to stand still. We must help our people, countries, and the world defend and attack this disease. Hence, we propose studying the use of body language using AI. We hope to collect and identify body parts’ gestures that characterize the epidemic in the upcoming studies on which we are currently working.

Table 1 indicates some studies that have used ML to discover disease and symptoms through gestures, hands, and facial expressions. This table concludes that the CNN algorithms are the most common and efficient methods of identifying disease symptoms through facial expressions and hand gestures. Some studies indicate that analyzing other body parts is also helpful in identifying some types of diseases using different ML algorithms, such as SVM and LSTM. It appears to us here that combining the proposed CNN algorithm with a new proposed algorithm to determine facial expressions will lead to high-quality results for detecting some epidemic diseases. It is essential first to study the symptoms that characterize the epidemic disease and their reflection on body expressions and then use the algorithm to learn the machine that has a higher efficiency in identifying these expressions.

The studies in Table 1 are classified as follows:

(1) Studies on medical diagnosis using AI for analyzing body language.
(2) Studies on medical diagnosis using electronic devices and AI for analyzing body language.
(3) Studies on COVID-19 diagnosis using other methods.

**Table 1:** Some Studies of AI Methods for Body Language Elements to Identify the Symptoms.

References	Title	Study Purpose	Method	Year	Result Evaluation	Future Work
[36]	Early prediction of disease progression in COVID-19 pneumonia patients with chest CT and clinical characteristics	Early recognition of COVID-19 Identify their treatment more individually Determine appropriate treatment	A retrospective multi-center application. Computed tomography (CT)	2020	Elevated NLR, Advanced age, and CT severity score are identified as separate risk factors for mild pneumonia COVID-19 clinical progression. The histogram shows desirable accuracy in the derivation and validation groups. CT scan of the chest can be used to predict disease risk severity and its progression risk.	
[37]	Individual-Level Fatality Prediction of COVID-19 Patients Using AI Methods	To predict if a patient with COVID-19 needs urgent health care Given the current pressure on limited medical resources.	A neural network is adopted. GitHub data processing. A Wolfram dataset. Logistic regression. Random forests, support vector machine (SVM). Local external factor. Isolation forest.	2020	To predict if a confirmed patient for COVID-19 is likely to die soon or not.	To create a model able to predict disease progression in addition to death. Prompting individuals to seek urgent care.
[38]	COVID-19 Prediction and Detection Using Deep Learning	To diagnose Coronavirus (COVID-19).	Deep convolutional neural network (DCNN). The system checks chest X-ray Prophetic Algorithm (PA) Long Short-Term Memory Neural Network (LSTM) Autoregressive integrated moving average (ARIMA).	2020	Forecast results show an accuracy of 94.80% and 88.43%	To investigate more complex forecasting methods.
[39]	Artificial Intelligence was applied to chest X-ray images to detect COVID-19 automatically. A thoughtful evaluation approach	To detect COVID-19 through the images of chest X-ray	A convolutional neural network (CNN). Three unique tests are performed following three pre-treatment plots. Survey what the preprocessing of the information means for the outcomes. A basic investigation is made of different issues of fluctuation.	2020	Classification accuracy of 91.5% is achieved.	
[40]	A Machine Learning Model to Identify Early-Stage Symptoms of SARS-CoV-2 Infected Patients	To distinguish the introduction highlights foreseeing Coronavirus infection determined to have high precision	A model that utilized supervised machine learning was created. The highlights analyzed incorporate age, sex, perception of fever, history of the movement, and clinical subtleties. XGBoost calculation.	2020	The highest accuracy in predicting and selecting features correctly (>85%). Frequent and critical prescient manifestations discovered are fever (41.1%), hack (30.3%), lung disease (13.1%), and runny nose (8.43%).	Utilizing a more extensive dataset will help in improving the ability to bypass these constraints and further improve prediction accuracy.
[41]	A combined deep CNNLSTM network for the detection of novel coronavirus (COVID-19) using X-ray image	To prevent COVID-19 from spreading Implementing it to automated machines.	CNN LSTM structure Utilizing Python and TensorFlow. Support vector machine (SVM) Random Forest	2020	The test result acquired 99.56% exactness and 80.53% recall for coronavirus cases. The system which recognized Coronavirus from chest X-beams got 95.2% precision, 100% specificity, and 93.3% affectability.	Develop combined CNN-LSTM. Detecting Coronvirus-19 using chest X-rays utilizing multiple datasets.
[42]	Smart and automation technologies for ensuring the long-term operation of a factory amid the COVID-19 pandemic: An evolving fuzzy assessment approach	To compare multiple applications of automated and intelligent technologies.	Both ACO and GA. MATLAB. FTOPSIS.	2020	Showing 93.48% accuracy, 98.75% sensitivity, and 92.85% specificity within the created system.	FWA will be connected to survey the execution of each automated application.
[43]	A Rapid, Accurate, and Machine-Agnostic Segmentation and Quantification Method for CT-Based COVID-19 Diagnosis	Proposing a fully automated, quick, precise, and machine-agnostic strategy.	creating a dynamic model. CT scanners Signal normalization. Breaking down the 3D division issue into three 2D issues,	2020	This model accomplished a categorization exactness of 89.5%. The worst-case error rate of this strategy is 4.9%. The worst-case error rate of other strategies is around the slightest 16%.	The learning stage can be utilized as a long-term caution framework for the long-run coronavirus.
[44]	Coronavirus (COVID-19) Classification using CT Images by Machine Learning Methods	To detect Coronavirus (COVID-19) disease.	Support Vector Machines (SVM). Local Directional Patterns (LDP). Grey Level Run Length Matrix (GLRLM). Discrete Wavelet Transform (DWT). Grey Level Co-occurrence Matrix (GLCM). Grey Level Size Zone Matrix (GLSZM).	2020	During a 10-fold cross-examination, the classification accuracy was still over 90 percent.	The machine learning approaches could be applied more to CT abdominal images, X-ray chest images, and blood test findings.
[45]	Early Prediction of Mortality Risk Among Severe COVID-19 Patients Using Machine Learning	To produce a clinical model to determine the result of critical COVID-19 patients earlier.	Logistic regression. Elastic net. Partial least squares regression. Bagged flexible discriminant analysis. Random forest. Recipient operating characteristic curve (AUROC).	2020	The AUROCs were 0.895 and 0.881, respectively. The sensitivity and accuracy were 89.2% and 68.7% for the source set and 83.9% and 79.4% for the validation set, sequentially, using a 50 percent death probability as the limit.	Constructing predictive models based on a larger sample size. Perform all Laboratory tests on all patients. Use patients who originate in the same hospital and have not transferred from other hospitals. Use patients in the best healthcare hospitals.
[46]	Multi-task deep learning-based CT imaging analysis for COVID-19 pneumonia: Classification and segmentation	To suggest a modern multi-task deep learning model.	A modern Multi-task Learning (MTL) design built on three errands Ordinary vs. COVID vs. Other Diseases classification COVID injury segmentation Picture remodeling.	2020	The enhancements are seen with an 88% dice coefficient for segmentation, which is 10% higher than when utilizing the condition of the U-net. With a sensitivity of 90.2% and a specificity of 99.7%. The categorization results have a 0.97 AUC and accuracy above 94%. The MTL model demonstrates a significant enhancement contrasted with different models producing results between 56% and 90%.	To research more recent sorts of networks while accounting for other valuable data and testing the method to affirm its performance using a larger dataset.
[47]	Artificial Intelligence and COVID-19: Deep Learning Approaches for Diagnosis and Treatment	To introduce deep learning techniques that are suitable for dealing with COVID-19 issues	ELM models. LSTM. The Recurrent Neural Network (Clockwork RNN (CW-RNN), GRURNN). The Generative Adversarial Network (GAN).	2020	Verify the accuracy and efficiency of deep learning techniques in diverse types of similar diseases.	Utilizing the proposed techniques to evaluate their effectiveness.
[38]	COVID-19 Prediction and Detection Using Deep Learning	To introduce an AI technique built on a deep convolutional neural network (CNN).	The framework examines chest X-ray pictures.	2020	The suggested system helps find COVID-19 and achieves an F-measure range of 95–99%.	The analysis of the COVID-19 spread and its related statistical data based on its global and regional distributions.
[48]	Automated detection and quantification of COVID-19 pneumonia: CT imaging analysis by a deep learning-based software	To employ deep learning-based software to assist in localization, recognition, and quantization of COVID-19.	An add up to 2460 RT-PCR tried COVID-19 positive.	2020	The standardized linear blended model demonstrated that the dorsal segment of the proper lower projection was the favored location of COVID-19 pneumonia.	Utilizing the proposed techniques to evaluate their effectiveness
[49]	Common cardiovascular risk factors and in-hospital mortality in 3894 patients with COVID-19: survival analysis and machine learning-based findings from the multicenter Italian CORIST Study	To recognize early COVID-19 symptoms exposing patients.	Review observational investigation on 3894 patients with SARS-CoV-2 disease in hospital. Random forest-based and Cox survival examination.	2020	(Hazard ratio (HR): 8.2; 95% certainty interval (CI) 4.6–14.7 for age ≥85 versus 18–44 y); HR = 4.7; 2.9–7.7 for assessed glomerular filtration rate levels <15 versus ≥90 mL/min/1. 73 m^2^; HR = 2.3; 1.5–3.6 for Creative protein levels ≥10 versus ≤3 mg/L).	Future correlation with the other Mediterranean and European Countries, conceivably including evaluations of different elements such as financial status.
[50]	InstaCovNet-19: A deep learning classification model for the detection of COVID-19 patients using Chest X-ray	Integrated stacked deep convolution network demonstrates using pre-trained models to cover a comparatively small sum of training data.	InstaCovNet-19 model. InceptionV3. MobileNet. NASNet.	2020	The average scores of the suggested model on ternary classification are 99%, 99%, and 99% for recall, precision, and F1, respectively. On the binary class, the model achieved 99% recall and 100% precision for the COVID class.	Deep learning procedures can likewise be applied to different manifestations of COVID-19, which present anomalies in the human organs.
[51]	Monitoring and analysis of the recovery rate of COVID-19 positive cases to prevent dangerous stages using IoT and sensors	To make a model forecast for COVID-19. To predict and dismember the apex speed of the contamination.	SVM. Bayesian algorithm. K-means algorithm.	2020	The prepared model shows a decent outcome in observing the therapy progress pace of the ailment.	Blend of other AI algorithms could be a decent wellspring for expanding the precision of the forecast
[52]	COVID-19 Patient Detection from Telephone Quality Speech Data	To explore the presence of signals around COVID-19 illness within the discourse data.	The dataset comprises discourse extricated from YouTube recordings, counting a video call of the COVID-19 patients and a video from a controlled studio environment. Each sentence is presented as super vectors of short-term Mel channel bank highlights for each phoneme. These features are utilized for training a two-class classifier; the classes are COVID-19 Positive and negative speakers.	2020	SVM classifier can accomplish an F1-Score of 92.7% and an accuracy of 88.6%.	To develop a fitting COVID-19 test based on telephone speech by acquiring more data since the study is limited to only 19 speakers. It will validate the results on the phoneme subclass level extensively.
[53]	Machine learning-based approaches to detect COVID-19 using clinical text data	To make a successful diagnosis of COVID-19.	Multinomial Naïve bayes (MNB). Support vector machine (SVM). Logistic regression. Decision tree. Adaboost. Random Forest. Bagging. Stochastic Gradient boosting for categorization	2020	Multinomial Naive Bayes has a precession of 94%, 95% f1 score, 96% recall, and an accuracy of 96.2% during testing. Gradient boosting and random forest obtained an accuracy of 94.3%.	Recurrent neural networks (RNN) can be utilized for better accuracy. They tend to use deep learning techniques in the future.
[54]	Data science and the role of Artificial Intelligence in achieving the fast diagnosis of COVID-19	To boost the speed of COVID-19 detection	Using AI with Chest X-rays and CT scans. Utilizing X-ray pictures and CT scan images with deep learning strategies.	2020	The recommended approach achieved 93% accuracy in CT scan images with a precision of 88% for chest X-ray images.	This research can be used to detect other diseases or infections.
[55]	DeepCOVIDNet: An Interpretable Deep Learning Model for Predictive Surveillance of COVID-19 Using Heterogeneous Features and Their Interactions	To predict the increasing range of future COVID-19 infections.	To compute equidimensional representations of novel methods of heterogeneous features such as multidimensional time-independent variables, multivariate spatial time series data, and multivariate time series.	2020	In about two and a half weeks), the average accuracy on those models is 63.7% upon using four output classes.	The deep FM design system can be changed to acquire the component associations. The creation of the deep FM structure can be adjusted to catch feature collaborations.
[56]	COVID-19 Detection Through Transfer Learning Using Multimodal Imaging Data	Coronavirus discovery utilizing pictures from the three most habitually used clinical imaging modes.	Convolutional Neural Network (CNN) models. VGG19 model.	2020	All lung picture modes produce around 100% for Ultrasound, 86% precision for X-ray, 84% for CT examines, and 100% for Ultrasound.	A study could isolate the lung field by segmenting all available image samples.
[57]	Early Detection of COVID19 by Deep Learning Transfer Model for Populations in Isolated Rural Areas	To utilize X-ray pictures in the early detection of COVID-19.	The processes from image pre-processing, data augmentation, VGG16, VGG19, and transfer of knowledge using DenseNet121 MobileNet Xception InceptionV3 InceptionResNetV2 networks Feature extraction ensemble classification as classifiers	2020	The accuracy of the various models ranged from 89.06% to 98%. VGG16 and VGG19 achieved the best accuracy of 96.88% and 95.31, respectively. After merging all the predicted classes of each model, the performance increases by showing the results of 98.66% precision, 98.33% recall, 98.30% F1-score, and 98% test accuracy.	Use more datasets from various sources to give more accurate and efficient results
[58]	Deep learning-based detection and analysis of COVID-19 on chest X-ray images	To use X-ray images in discovering methods for classifying COVID-19.	Deep learning-based CNN models (Xception, ResNet, and Inception V3 models) were utilized.	2020	The best accuracy of 97.97% was obtained from the Xception model.	The accuracy and performance of the work can be validated by having a larger dataset for chest X-rays.
[59]	Analysis of novel coronavirus (COVID-19) using machine learning methods	To study the COVID-19 data to analyze the spreading factors.	Support Vector Regression (SVR) to do classification and clustering of data.	2020	Results on average accuracy evaluation for the total number of cases across all four countries: Simple Linear Regression: 75.64% Polynomial Regression: 98.01% Support Vector Regression: 99.18% For the average growth rate spread across all four countries: Simple Linear Regression:66.12% Polynomial Regression: 92.04% SVR: 99.19% Average prediction score across all four countries: Simple Linear Regression: 72.89% Polynomial Regression: 99.32% SVR: 99.18%.	Combining another AI Algorithm such as Neural Networks could improve the model of the dataset.
[11]	Facial emotion detection using deep learning	To determine basic facial emotions	Deep learning methodologies are used on the images. Applying the pure convolutional neural network method	2016	The results are not up to date but slightly better than those using other technologies, including feature engineering.	A more extensive data set can improve network performance.
[23]	Measuring facial expressions of emotion	Determining facial expression of emotions Understanding emotions in people with mental illness.	The three approaches of emotion facial action coding system Electromyography automatic face is used to measure the facial expression of emotion	2007	Measuring and describing facial expressions of feelings improves understanding and intervention in people with mental illness.	To produce more innovative studies in the facial expression of emotions that may give detailed answers to questions that are not yet resolved in emotion research.
[60]	PRATIT: a CNN-based emotion recognition system using histogram equalization and data augmentation	To classify facial expressions according to seven basic emotions.	PRATIT is used to recognize facial expressions. Pre-processing procedures such as Grayscale Resizing Cropping Histogram equalization are utilized to address image differences.	2020	The model achieves a training accuracy of 95.1%. Cross-checks the test accuracy of 64.69%. A test accuracy of 64.62%, excluding graph equalization. More data increase the verification accuracy to 76.01% and the test accuracy to 76.03%. Resulting in an improved verification accuracy of 79.19% compared to the test accuracy of 78.52% of other methods.	To measure the model’s performance using photos from various datasets and sources to perform various experiments.
[61]	Facial expression video analysis for depression detection in Chinese patients	To analyze the emotional state of facial expressions to identify emotions.	Feature classification is done using SVM	2018	The achieved detection accuracy rate is 78.85%, and recall is 80.77%.	To address additional factors that affect agitation and retardation to improve the accuracy of depression detection.
[62]	A Novel Facial Thermal Feature Extraction Method for Non-Contact Healthcare System	To measure face temperature electronically as a diagnostic tool.	A classic CNN is run under the DIGITS platform using CAFFE, Google Net. Four models are trained using RGB raw image RGB feature image Thermal raw image Thermal feature image.	2020	Feature images achieved the highest prediction resolution.	To enable long-term health tracking by extending the proposed method.
[63]	Emotion Detection Using Facial Recognition	To detect emotions through facial expressions.	Detect emotions using facial expressions with a convolutional neural network (CNN). Emotion detection experiments are performed using FER2013 JAFFE datasets.	2020	The highest F1 score for detecting feelings is 77.34 for happy emotions for the FER2013 data set. 75.50 for sudden emotion using the JAFFE dataset.	To extend facial recognition systems based on Principal Component Analysis (PCA) Independent Component Analysis (ICA).
[64]	Stress and anxiety detection using facial cues from videos	To detect and analyze emotional states of anxiety through videotaped facial signals.	An element choice methodology is utilized to recognize the heartiest qualities, followed by arrangement plots that separate between pressure and nonpartisan states.	2017	Accomplish great and suitable exactness as unfair markers of stress and nervousness.	To add video recordings of at least one-minute duration.
[65]	Automatic Detection of ADHD and ASD from Expressive Behavior in RGBD Data	To decide symptomatic forecasts about the occupancy of ADHD and ASD.	Selecting members utilizing current RGBD (color + depth) sensors.	2017	Classification rate is 96% for the controls vs. condition (ADHD/ASD) gatherings. 94% for the Comorbid gathering (ADHD + ASD) versus just ASD	
[66]	A Facial-Expression Monitoring System for Improved Healthcare in Smart Cities	To recognize facial expressions to improve healthcare service in the smart city.	The CS-LBP histograms of the blocks are joined to create a vector of the facial picture. The discretionary feature determination procedure characterizes the largest predominant highlights. Then, applied to a Gaussian mixture model and a supporting vector machine.	2017	The stated framework can perceive facial expressions showing 99.95% exactness.	To incorporate electronic medical patient’s history into the system.
[67]	Deep Pain: Exploiting Long Short-Term Memory Networks for Facial Expression Classification	To assess pain in patients to see recovery through facial features.	Convolutional neural organizations (CNNs) have been utilized. Take in facial features from VGG_Faces. Then, connected to long short-term memory to harness the temporal connection within video frames.	2017	Obtained an AUC value of 89.6, an increase of up to 93.3 when using the same CNN with feature extraction for RNN training. 97.2% accuracy was obtained on the facial emotion recognition data set.	
[68]	Patient State Recognition System for Healthcare Using Speech and Facial Expressions	To recognize the patient status of the healthcare.	The system utilizes two sorts of input (audio and video) caught in a multisensory climate.	2016	The proposed system achieves an average recognition accuracy of 98.2%.	To improve the layout and reduce system time using features with fewer dimensions.
[69]	Facial expression monitoring system for predicting patient’s sudden movement during radiotherapy using deep learning	To monitor a subject’s facial expressions and predict their movement during treatment.	A convolutional neural model and expanded Cohn-Kanade dataset.	2020	The accuracy of training and testing is 100% and 85.6%.	
[70]	Patient Monitoring System from Facial Expressions using Python	For automatic detection of pain by facial expressions.	A framework for facial expression recognition with the usage of captured photos. A framework utilizes the CNN classifier to separate the procured picture of various emotion classifications.	2020	The dataset is already developed with 92.4% accuracy.	
[71]	Combining Facial Expressions and Electroencephalography to Enhance Emotion Recognition	To recognize emotions by compounding EEG and facial expressions.	An information base for investigating emotions utilizing Physiological Signs (DEAP) and a MAHNOB human–computer interface (MAHNOB-HCI) are utilized for assessment.	2019	An accuracy of 69.75% is accomplished for the parity space 70.00% for the excitation space after combination. Each surpasses the most noteworthy performing single technique 69.28% for parity space. 64.00% for excitation space.	To gather additional EEG data or generate data using a generative paradigm of quasi-supervised learning.
[72]	Gestures Controlled Audio Assistive Software for the Voice Impaired and Paralysis Patients	To help patients with severe cerebral palsy and voice impairment.	Made with Microsoft Visual Studio 2015 (Free Stage) open source.	2019	Disabled patients can run the program effectively and efficiently	
[73]	Detecting speech impairments from temporal Visual facial features of aphasia patients	To discover speech impairment from videos of people with aphasia.	A cross-media approach is proposed utilizing visual facial highlights to find discourse attributes without thinking about the phonemic substance of discourse.	2019	The improved features can differentiate with a precision of 0.86. A blend of these features improves the performance accuracy to 0.88.	To incorporate body movement into the study to discover gestures as a surrogate for lost speech abilities.
[74]	Gestures Controlled Audio Assistive Software for Voice Impaired and Paralysis Patients	To estimate schizophrenia symptoms by the automatic analysis of facial expressions.	SchiNet is proposed as a novel neural network design approximating articulation-related symptoms in two distinctive assessment interviews for the patient-autonomous forecast of schizophrenia manifestations.	2019	The proposed network for assessing indication seriousness offers promising outcomes.	To improve the exhibition of AFEA through temporal investigation. Stretch out the conduct examination to incorporate body motions and vocal articulations.
[75]	Detecting Speech Impairments from Temporal Visual Facial Features of Aphasia Patients	To recognize hand gestures as an aid to patient care.	The framework utilizes Haar-like elements, Cascade classification for hand detection, and the Adaboost algorithm.	2018	The accuracy for hand detection was 91.88%. The sensitivity is above 95.75%.	It is suggested to try out the proposed work for reality.
[22]	The Elements of End-to-end Deep Face Recognition: A Survey of Recent Advances	Using appropriate methods to build a state-of-the-art end-to-end face recognition system from scratch.	They concentrate on end-to-end deep face recognition based on 2D pictures. Taking generic photographs or video frames as input and extracting the in-depth features of each face as output. Face detection, face alignment, and face representation are the three fundamental parts of an end-to-end deep face recognition system.	2021	To determine which approach is a strong-baseline style for comparison in the experiment. To examine current issues and identify certain interesting future research topics.	
[76]	Automated Facial Action Coding System for Dynamic Analysis of Facial Expressions in Neuropsychiatric Disorders	They created a cutting-edge automated FACS system to assess dynamic changes in facial motions.	The automated FACS system and its application to video analysis will be described. processed for feature extraction Image Processing Action Unit Detection Application to Video Analysis	2011	Controls 3, 2, and 4 were extremely expressive (flatness = 0.0051, 0.0552, 0.1320) Patients 1, 2, and 4 were very flat (flatness = 0.8336, 0.5731, 0.5288) Control 1 and patient 3 had flatness values of 0.3848 and 0.3076, respectively Patients 4 and 3 had the most improper expression (inappropriateness = 0.6725, 0.3398, 0.3150) Patient 1 and controls 1–4 had the least (inappropriateness = 0.2579, 0.2506, 0.2502, 0.1464, 0.0539)	
[23]	Measuring facial expression of emotion	To discuss three ways to evaluate facial expressions of emotion and their contributions.	Based on Ekman’s EMFACS-System. SHORETM’s ability to work in real-time with Google Glass is an excellent feature.	2015	This scenario may change since several technologies for completely automatic face recognition are now commercially accessible and generate sufficient reliable data.	
[10]	Classifying Facial Action	To determine the face measure and muscle contractions involved in facial expression.	Three methods are compared classify facial expressions combine them to identify the best performance.	1996	The methods achieved 89%, 57%, and 85%, respectively. The combined method achieves 92%.	To apply these techniques to lower facial movements to obtain a fully automated method.
[24]	Real-Time Gait Analysis Algorithm for Patient Activity Detection to Understand and Respond to the Movements	To discover the vibrational movement of patients with neurological disorders.	An automated file-based real-world data is developed to monitor and detect gait deviation.	2012	Easy to develop and can effectively detect patient’s limb movements and generate health care alerts.	To detect epileptic seizures. To alert healthcare workers about an ongoing emergency.
[25]	A Real-Time Patient Monitoring Framework for Fall Detection	To develop an effective system that predicts the imminent fall of elderly patients.	Using MbientLab sensors. The model architecture uses a long short-term memory (LSTM) neural network. For data analytics flow, Apache Flink is employed. Mobi Act dataset is used to train the model	2019	The proposed model achieves drop detection with 95.8% accuracy.	To expand the framework by supporting various sensors, enabling parallel data processing pipelines, and cloud integration.
[26]	Anomaly Detection of Elderly Patient Activities in Smart Homes using a Graph-Based Approach	To discover and predict the behavior of elderly patients to improve their home safety.	A graph-based approach is used. The smart home daily activity diagrams are analyzed to reveal standard patterns and spatial, temporal, and behavioral abnormalities.	2018	Using a graph-based approach based on data collected on the residents’ activities.	To expand the experiments by including real-time data flow. Transforming real-time sensor records for real-time health monitoring.
[28]	Fall Detection and Activity Recognition with Machine Learning	To prevent the elderly from falling and other health problems.	The users are equipped with radio cards to determine the distinct areas. Eight machine learning algorithms that support machine vectors are compared to identify the most accurate classifier.	2008	The highest rating accuracy of 95% is achieved.	To adjust and augment the machine learning algorithms.
[30]	Vision-based detection of unusual patient activity	To analyze the behavior of psychiatric patients. Using monitoring to reduce the risks of harm.	The patients are monitored using surveillance cameras. Luminous flux vector statistics are extracted from the patient’s movements to determine risky behavior.	2011	The initial result indicates that the system can be applied in a real hospital scenario to prevent injuries to patients and staff.	To obtain a more comprehensive data set for a more comprehensive statistical evaluation of the result.
[31]	MDS: Multi-level decision system for patient behavior analysis based on wearable device information	To monitor patients, facilitate diagnosis, and predict the disease.	A multi-level decision system (MDS) is used. The information is matched with historical data to determine the patient’s current state of health.	2019	Improved true positive rate, accuracy, F-score, and reduced fusion delay.	To widen the scope of decision-making and include other activities for different age groups.
[77]	Proposal Gesture Recognition Algorithm Combining CNN for Health Monitoring	To process patient data for treatment systems and self-learning.	The UCF101 dataset features different activities. Three videos (baby walking, walking, falling) track and monitor infants or older adults. A long short-term memory (LSTM) model is used. Using convolutional neural networks (CNN).	2019	The display resolution is 99%.	Using CNN models for real application tagging on wireless sensor networks (WSNs) and SSDLite Mobile net overflow tensioner to define actions and create functionality on Android; suitable for smart home apps.
[78]	A robust method for VR-based hand gesture recognition using density-based CNN	To help patients with impaired mobility due to accident, disease, or another injury.	The data set is retrieved from VR sets such as HTC Vive and leap motion. It is represented as 2D images and consists of 14 types of hand motions. Using 33 mapped 3D points in binary images as input. The proposed density-based CNN is trained with the input characteristics.	2020	The feature extraction fraction and classification layer using loss function SoftMax achieves 97.7% accuracy.	To apply a transfer learning scheme, maintain CNN fragment weight, and retrain only fully connected, density-based CNN layers using pyramid core size compatible with similar datasets.
[79]	Hand Gesture Recognition Using Convolutional Neural Network for People Who Have Experienced a Stroke	To identify hand gestures for deaf patients and stroke survivors.	A system for identifying hand movement based on a convolutional neural network (CNN).	2019	The accuracy of the test is up to 99% using CNN.	To increase the number of common gestures to ten using a 3D endoscopic camera.
[80]	Determining the affective body language of older adults during socially assistive HRI	To know and control the appropriate times.	A 3D data system is built From the KinectTM sensor to the robot to identify Body language features using a person’s 3D skeleton upper body.	2014	Elderly users show affective states, which further motivates the use of the affect rating system.	Investigating the multimedia effect from a set of body language, voice tones, and facial expressions.
[81]	Vision based body gesture meta features for Affective Computing	For the early discovery of psychological distress through body language.	Highlight assessment is removed from recordings, motion recognition inside body parts, and meta-data from singular signals. These highlights are consolidated into a short element vector for use in expectation assignments. Another informational collection of 65 video accounts of meetings with self-surveyed pain and character.	2020	Within the new dataset, An F1 score of 82.70% was achieved for predicting depression.	To expand the presented data set and increase its quality via manual annotations.
[82]	Deep Learning and Medical Diagnosis: A Review of Literature	To determine whether a deep learning application is helpful in medical diagnostics.	Over 300 research articles have been identified, and 46 articles submitted for a detailed review.	2020	Convolutional neural networks (CNNs) are overrepresented in medical image analysis.	To conduct a more rated review and add deep learning development and application during specific periods.
[83]	Machine learning classification of design team members’ body language patterns for real time emotional state detection	To detect the emotional state of team members individually and automatically using non-wearable sensors.	A machine-learning-driven methodology (C4.5, Random Forest, IBk, and Naive Bayesian) is suggested to identify individual emotional states using non-wearable, low-cost sensors in the design team.	2015	To detect emotional states with an accuracy of over 98%.	Not to limit the methodology to modeling individual members of the team’s emotional state
[84]	Towards Automatic Detection of Amyotrophic Lateral Sclerosis from Speech Acoustic and Articulatory Samples	To automatically detect amyotrophic lateral sclerosis (ALS).	Automatic detection of ALS is achieved from the short and pre-symptomatic vocals. Using machine learning methods (deep neural networks and SVMs)	2016	The addition of articular movement information (from lips and tongue) improves detection performance.	To use a more extensive data set to validate our approach for detecting ALS from speech samples.
[85]	Towards improving diagnosis of skin diseases by combining deep neural network and human knowledge	To diagnose skin disease using human knowledge and deep neural network.	Using a deep learning algorithm to diagnose skin disease from common cutaneous diseased. Several images with multiple semantic hierarchical structures of skin disease were used to enhance the algorithm’s accuracy.	2018	Dataset shows 87.25% accuracy meanwhile 86.63% for dataset B. The standard deviation of the result is 2% to 5%, reflecting the accuracy variation.	Enhanced image detection or using only clean images can improve the reading accuracy of the algorithm.
[86]	Early prediction of chronic disease using an efficient machine learning algorithm through adaptive probabilistic divergence-based feature selection approach	To predict the accuracy of the sickness’s ebb and flow phase period.	To execute a model through an AI algorithm on the identification of early chronic sickness	2020	Precision of 91.6 was enrolled during the time spent on the CKD sickness forecast.	
[87]	Multi-Modal Depression Detection and Estimation	To improve depression discovery/estimation performance. To produce depression information.	Suggest various unique techniques, which are Novel FACS 3D and Generative Adversarial Network (GAN)	2019	DCGAN-based data generation approach successfully moves forward the performance of depression estimation.	Attempts to zero in on joining depression estimation with dimensional emotional analysis.
[88]	Dual-hand detection for human-robot interaction by a parallel network based on hand detection and body pose estimation	To detect hand movements for posture information.	A deep parallel neural network uses two channels: The first channel uses the ResNet–Inception–Single Shot MultiBox detector to obtain information to identify hand features. The second channel detects the position of the human body followed by the positioning of the left and right by revealing the physical skeleton through the forward movement.	2019	The results indicate that the proposed deep parallel neural network accurately identifies the features of both hands.	It is suggested that the astronaut use the method to assist robots in interacting and understanding hand movements.
[89]	Gesture recognition based on multi-modal feature weight	To identify gestures for depth and red, green, and blue (RGB) images.	According to the weight of the adaptive fusion multimodal. A multimodal adaptive fusion method was used to weigh	2021	Simulation experiments show that: The proposed method is better than the traditional RGB-D gesture image processing method. Its gesture recognition rate is higher.	It is recommended to discuss the influence of different network structures for this method.
[90]	Pose-based Body Language Recognition for Emotion and Psychiatric Symptom Interpretation	To provide an automated system for body language-based emotion recognition based on RGB videos.	In the first step, the model takes the pose sequence p as input and produces two body language sequences that reflect upper- and lower-body body language, respectively. The second level learns the emotions conveyed by the individual of interest using the two anticipated body language sequences as inputs.	2021	When just 20% of the training data is utilized, RGB-based algorithms perform worse (86.0 percent 65.0 percent)/86.0 percent = 24.4 percent, whereas ST-Convpose performs better (15.7 percent). When ST-Convpose is pretrained on NTU RGB + D [48] of 5.2 percent, the performance reduction is significantly lower. The ST-ConvPose works effectively with a small quantity of training data.	

This study aims to survey research using ML algorithms to identify body features, movements, and expressions. Each movement is affected by the disease, and each disease is characterized by a distinct and different effect on the body. This means some body parts will undergo certain changes that point to a specific disease. Thus, we propose that ML algorithms capture images of body movements and expressions, analyze them, and identify diseases. This study surveyed a selection of existing studies that use different ML algorithms to detect body movements and expressions. Since these studies do not discuss this epidemiology method, this study seeks to document the use of ML algorithms in discovering epidemics such as COVID-19. Our survey analysis concludes that the results achieved indicate the possibility of identifying the body movements and expressions and that ML and convolutional neural networks are the most proficient in determining body language.

From an epidemiological, diagnostic, and pharmacological standpoint, AI has yet to play a substantial part in the fight against coronavirus. Its application is limited by a shortage of data, outlier data, and an abundance of noise. It is vital to create unbiased time series data for AI training. While the expanding number of worldwide activities in this area is promising, more diagnostic testing is required, not just for supplying training data for AI models but also for better controlling the epidemic and lowering the cost of human lives and economic harm. Clearly, data are crucial in determining if AI can be used to combat future diseases and pandemics. As [91] previously stated, the risk is that public health reasons will override data privacy concerns. Long after the epidemic has passed, governments may choose to continue the unparalleled surveillance of their population. As a result, worries regarding data privacy are reasonable.

## 7. Conclusions

According to patient surveys, communication is one of the most crucial skills a physician should have. However, communication encompasses more than just what is spoken. From the time a patient first visits a physician, their nonverbal communication, or “body language”, determines the course of therapy. Bodily language encompasses all nonverbal forms of communication, including posture, facial expression, and body movements. Being aware of such habits can help doctors get more access to their patients. Patient involvement, compliance, and the result can all be influenced by effective nonverbal communication.

Pandemic and epidemic illnesses are a worldwide threat that might kill millions. Doctors have limited abilities to recognize and treat victims. Human and technological resources are still in short supply regarding epidemic and pandemic conditions. To better the treatment process and when the patient cannot travel to the treatment location, remote diagnosis is necessary, and the patient’s status should be automatically examined. Altering facial wrinkles, movements of the eyes and eyebrows, some protrusion of the nose, changing the lips, and the appearance of certain motions of the hands, shoulders, chest, head, and other areas of the body are all characteristics of pandemic and epidemic illnesses. AI technology has shown promise in understanding these motions and cues in some cases. As a result, the concept of allocating body language to identifying epidemic diseases in patients early, treating them before, and assisting doctors in recognizing them arose owing to the speed with which they spread and people died. It should be emphasized that the COVID-19 disease, which horrified the entire world and revolutionized the world’s life, was the significant and crucial motivator for the idea of this study after we studied the body language analysis research in healthcare and defined the automatic recognition frame using AI to recognize various body language elements.

As researchers in information technology and computer science, we must contribute to discussing an automatic gesture recognition model that helps better identify the external symptoms of epidemic and pandemic diseases to help humanity.

## Data Availability

Not applicable.
